# Was ist gesichert in der Therapie der chronischen Nierenerkrankung?

**DOI:** 10.1007/s00108-022-01422-9

**Published:** 2022-11-02

**Authors:** Robert Greite, Kai Schmidt-Ott

**Affiliations:** Klinik für Nieren- und Hochdruckerkrankungen, Medizinische Hochschule Hannover (MHH), OE 6840, Carl-Neuberg-Str. 1, 30625 Hannover, Deutschland

**Keywords:** Albuminurie, Chronische Nierenerkrankung/Progression, Natrium-Glukose-Kotransporter-2-Inhibitoren, Finerenon, Glucagon-like-peptide-1-Rezeptor-Agonisten, Albuminuria, Kidney disease, chronic/progression, Sodium-glucose transporter 2 inhibitors, Finerenone, Glucagon-like peptide 1 receptor agonists

## Abstract

Man spricht von einer chronischen Nierenerkrankung („chronic kidney disease“ [CKD]), wenn über einen längeren Zeitraum (≥ 3 Monate) eine reduzierte glomeruläre Filtrationsrate (GFR) oder eine relevante Albuminausscheidung im Urin beobachtet wird. Die Ursachen von CKD sind vielfältig, wobei die Assoziation mit Diabetes mellitus am häufigsten ist. Frühe Stadien von CKD betreffen etwa 10 % der Gesamtbevölkerung. Mit Abnahme der GFR und Zunahme der Albuminurie steigen die Häufigkeit kardiovaskulärer Ereignisse, das Risiko einer Dialysepflichtigkeit und die Gesamtmortalität exponentiell an. Die Leitlinien der Deutschen Gesellschaft für Allgemeinmedizin und Familienmedizin (DEGAM) und der Organisation Kidney Disease: Improving Global Outcomes (KDIGO) empfehlen eine Überweisung in die Nephrologie bei einer GFR ≤ 30 oder ≤ 60 ml/min pro 1,73 m^2^ bei Vorliegen verschiedener Begleitfaktoren. Das bedeutet, dass ein Großteil der CKD-Patienten allgemeininternistisch oder hausärztlich behandelt wird. Im vorliegenden Beitrag möchten wir die Datenlage zur Behandlung von CKD und deren Komplikationen in der Praxis komprimiert zusammenfassen. Wir gehen dabei auf aktuelle Leitlinienempfehlungen ein, diskutieren aber auch neue Studienergebnisse, die perspektivisch das therapeutische Repertoire erweitern könnten.

## Abstract (en)

Chronic kidney disease (CKD) is defined as a relevant excretion of albumin into the urine or a reduction of the glomerular filtration rate (GFR) over a longer time period of ≥ 3 months. The causes of CKD are manifold, whereby the association with diabetes mellitus is the most frequent cause. Early stages of CKD affect approximately 10% of the total population. The frequency of cardiovascular events, the risk of dependency on dialysis and the all-cause mortality increase exponentially with a decrease in the GFR and an increase in albuminuria. The guidelines of the German College of General Practitioners and Family Physicians (DEGAM) and the organization Kidney Disease: Improving Global Outcomes (KDIGO) recommend referral to a nephrologist with a GFR of ≤ 30 or ≤ 60 ml/min/1.73 m^2^ in the presence of various cofactors. This means that the majority of CKD patients are treated by general internists or general practitioners. This article gives a concise summary of current data on the treatment of CKD and its associated complications in clinical practice. It refers to the current guidelines and also new study results which could perspectively expand the therapeutic repertoire.

Die chronische Nierenerkrankung („chronic kidney disease“ [CKD]) ist häufig und verläuft oft progredient [1]. Am Ende der Erkrankung können Dialyse oder Nierentransplantation notwendig werden, jedoch steigt die Mortalität schon vorher mit abnehmender Nierenfunktion und zunehmender Eiweißausscheidung rasant an [2]. Dabei stehen kardiovaskuläre Todesursachen im Vordergrund. Die Progression kann allerdings verlangsamt werden.

Bei der Therapie der CKD gibt es entscheidende Neuerungen. Neben den etablierten Therapien mit Inhibitoren des „angiotensin-converting enzyme“ (ACEi) oder Angiotensinrezeptorblockern (ARB) stellt die kürzliche Zulassung eines Inhibitors des Natrium-Glukose-Kotransporters 2 („sodium-glucose co-transporter 2“) [SGLT2] zur CKD-Therapie einen Meilenstein dar. Umfangreiche klinische Studien legen nahe, dass diese Substanzklasse die Gesamtmortalität und das Risiko einer CKD-Progression in unterschiedlichen klinischen Situationen deutlich reduziert. Bei CKD mit Diabetes kommen weitere Optionen hinzu: So konnten für Glucagon-like-peptide-1-Rezeptor-Agonisten (GLP-1-RA) und für einen nichtsteroidalen Mineralokortikoidrezeptorantagonisten (MRA) positive Daten hinsichtlich einer CKD-Progressions-Hemmung gezeigt werden. Im vorliegenden Beitrag geben wir einen Überblick über ärztliche Entscheidungsprozesse in der CKD-Versorgung und legen einen Fokus auf hausärztlich und allgemeininternistisch relevante Aspekte.

## Definition und Diagnostik der chronischen Nierenerkrankung

**Figure 1:**
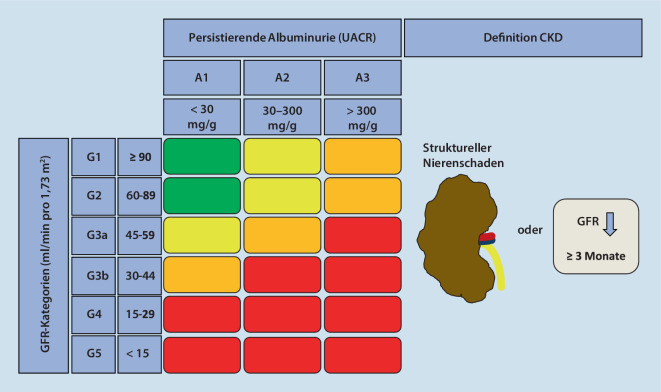

Die CKD ist definiert als eine Veränderung der Nierenstruktur oder Einschränkung der Nierenfunktion, die länger als 3 Monate besteht (Abb. 1). Zur Ermittlung der Nierenfunktion wird die Bestimmung des Serumkreatinins und Abschätzung der glomerulären Filtrationsrate (GFR) anhand der Chronic-Kidney-Disease-Epidemiology-Collaboration(CKD-EPI)-Formel empfohlen [3]. Entscheidend ist jedoch, dass zur vollständigen Diagnostik und Stadieneinteilung von CKD auch die Bestimmung der Albuminausscheidung im Urin relevant ist. Hierzu werden Albumin und Kreatinin im Spontanurin bestimmt und als Verhältnis angegeben („urinary albumin-creatinine ratio“ [UACR]; [3]). Eine Urinsammlung ist hierfür nicht erforderlich. Es ist wichtig, dass die UACR auch bei völlig normaler GFR pathologisch sein und die Diagnose einer CKD anzeigen kann. Somit ist es in der internistischen und allgemeinärztlichen Praxis entscheidend, regelmäßige UACR-Bestimmungen durchzuführen, um relevante und therapiebedürftige CKD-Formen nicht zu übersehen.

Die Organisation Kidney Disease: Improving Global Outcomes (KDIGO) empfiehlt in ihrer Leitlinie die Klassifikation der CKD anhand der Ursache sowie nach 6 GFR- und 3 Albuminurie-Kategorien ([3]; Abb. 1). Für die Prognose ist die Höhe der Albuminurie ein entscheidender Faktor. Während Patienten mit einer GFR > 60 ml/min pro 1,73 m^2^ ohne pathologische Albuminausscheidung ein normales Mortalitätsrisiko haben, ist es bei Patienten mit der gleichen Nierenfunktion, aber einer Albuminurie von ≥ 300 mg/g bereits 3‑fach erhöht [2]. Sinkt die GFR unter 60 ml/min pro 1,73 m^2^, steigt das Mortalitätsrisiko exponentiell an [2].

## Betreuung von CKD-Patienten in der Hausarztpraxis und Überweisung in die Nephrologie

**Figure 2:**
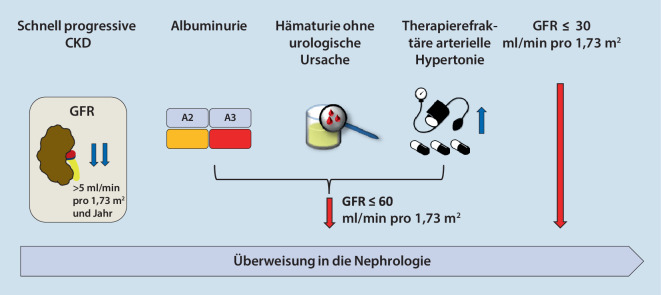

In der 2019 erschienenen S3-Leitlinie der Deutschen Gesellschaft für Allgemeinmedizin und Familienmedizin (DEGAM) zur Versorgung von Patienten mit chronischer nichtdialysepflichtiger Nierenerkrankung in der Hausarztpraxis [4] werden als Kriterien für die Überweisung in die Nephrologie (Abb. 2) eine Reduktion der GFR auf ≤ 30 ml/min pro 1,73 m^2^ oder auf ≤ 60 ml/min pro 1,73 m^2^ genannt, wenn begleitend eine Albuminurie ≥ Stadium A2, eine nicht urologisch erklärbare Hämaturie, ein therapierefraktärer arterieller Hypertonus oder eine schnell voranschreitende CKD vorliegt. Eine rasche Progression ist dabei definiert als Abnahme der GFR um ≥ 5 ml/min pro 1,73 m^2^ pro Jahr. Diese Kriterien entsprechen auch der KDIGO-Leitlinie ([3]; Abb. 2).

## Management der chronischen Nierenerkrankung

**Figure 3:**
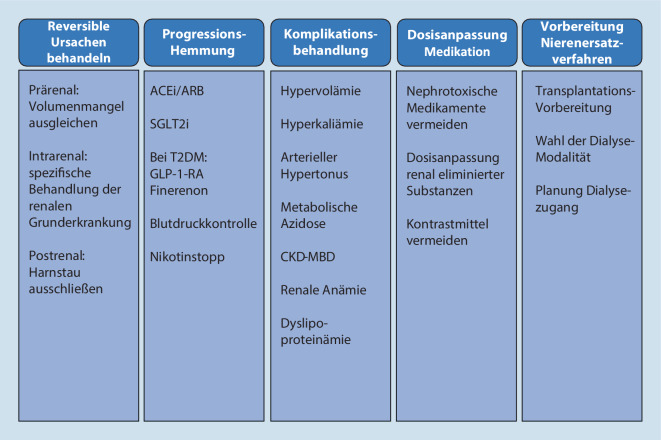

In Abb. 3 sind die verschiedenen Aspekte des Managements von Patienten mit CKD übersichtlich zusammengefasst. Diese Aspekte werden im Folgenden erläutert.

### Erkennung und Behandlung reversibler Ursachen einer Funktionsverschlechterung bei chronischer Nierenerkrankung

Grundsätzlich sollten Patienten mit CKD bei neu aufgetretener Funktionsverschlechterung auf reversible Ursachen untersucht werden. Dabei ist neben dem Ausgleich eines Volumenmangels und dem Ausschluss einer postrenalen Komponente die möglichst spezifische Therapie der renalen Grunderkrankung (beispielsweise Immunsuppression bei autoimmunen Nierenerkrankungen) wichtig.

### Progressionshemmung

**Figure 4:**
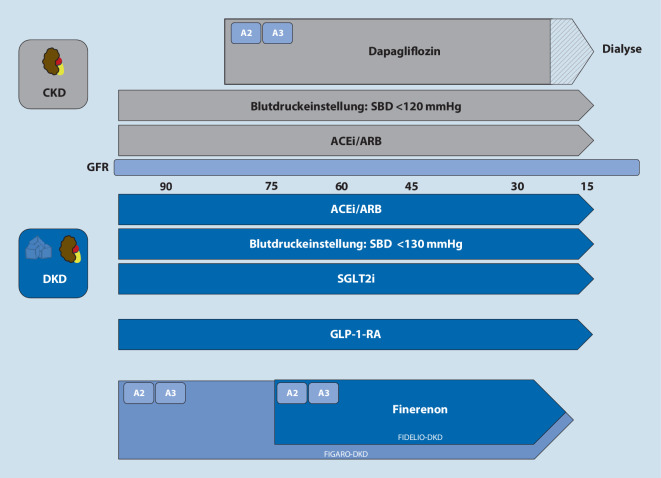

Das Voranschreiten einer CKD zu verhindern, ist essenziell für die Gesamtprognose. Hierfür gibt es medikamentöse und nichtmedikamentöse Ansätze. Vor allem bei den medikamentösen Ansätzen gab es in den letzten Jahren entscheidende Neuerungen. Im Folgenden stellen wir die medikamentösen Ansätze dar, die bei der CKD-Progressions-Hemmung eine Rolle spielen (Abb. 4).

#### Medikamentöse Progressionshemmung mit ACE-Inhibitoren oder Angiotensinrezeptorblockern

ACEi oder ARB sind die antihypertensiven Therapeutika der Wahl bei CKD und werden in der aktuellen KDIGO-Leitlinie für alle Patienten mit einem systolischen Blutdruck (SBD) > 120 mm Hg in den CKD-Stadien G1–G4 und A2–A3 mit oder ohne Diabetes mellitus empfohlen [5]. Unabhängig von der Blutdrucksenkung ist der nephroprotektive Effekt von ACEi/ARB bei CKD durch Senkung der Proteinurie gut gesichert [6]. Eine Kombination von ACEi und ARB zur Senkung der Proteinurie hingegen ist aufgrund relevanter Sicherheitsbedenken nicht zu empfehlen [7].

### Therapie von Patienten mit chronischer Niereninsuffizienz und Diabetes mellitus Typ 2

#### SGLT2-Inhibitoren

In der KDIGO-Leitlinie zum Management von Diabetes mellitus bei CKD von 2020 wird die Gabe von SGLT2-Inhibitoren (SGLT2i) neben Lifestyle-Modifikation und Metformin mittlerweile als Erstlinientherapie des Diabetes mellitus Typ 2 (T2DM) empfohlen [8]. Die Leitlinie empfiehlt dabei den Beginn einer Therapie mit SGLT2i, wenn die GFR > 30 ml/min pro 1,73 m^2^ liegt, und das Absetzen bei Erreichen der Dialysepflichtigkeit [8].

#### GLP-1-Rezeptor-Agonisten

Sollten durch die Erstliniendiabetestherapie nicht die individualisiert empfohlenen Hämoglobin-A1c(HbA1c)-Werte von 6,5 bis 8,0 % erreicht werden, empfiehlt die Leitlinie eine Ergänzung um einen lang wirksamen GLP-1-RA [8]. Für GLP-1-RA ist neben einer Senkung der kardiovaskulären Mortalität auch eine Hemmung der CKD-Progression mit Senkung der Albuminurie und des GFR-Abfalls nachgewiesen [9].

#### Finerenon

Kürzlich wurde mit Finerenon eine vielversprechende Substanz zur Behandlung von Patienten mit CKD und T2DM in der Europäischen Union (EU) zugelassen. Finerenon ist ein MRA, allerdings anders als Spironolacton oder Eplerenon nichtsteroidal und offenbar mit einem niedrigeren Hyperkaliämierisiko behaftet [10]. Grundlage für die EU-Zulassung bildeten die Daten der FIDELIO-DKD- und FIGARO-DKD-Studien.

In FIDELIO-DKD wurden 5734 Patienten mit CKD und T2D zu Finerenon oder Placebo randomisiert. Eingeschlossen wurden hierbei Patienten mit einer GFR zwischen 25 und 75 ml/min pro 1,73 m^2^ und einer UACR > 30 mg/g und ≤ 5000 mg/g. Wichtig ist, dass alle Patienten eine Standardtherapie zur Blutzuckersenkung und die maximal tolerierte ACEi/ARB-Dosis erhielten. Das Risiko von kardiovaskulärer Mortalität und CKD-Progression war nach 2,6 Jahren in der Finerenongruppe signifikant reduziert [11]. Die 2021 erschienene FIGARO-DKD-Studie untersuchte ergänzend zu FIDELIO-DKD einen kombinierten kardiovaskulären Endpunkt. In FIGARO-DKD wurden auch Patienten mit niedrigeren CKD-Stadien (bis G1), aber schwerer Albuminurie eingeschlossen. Bei diesen Patienten reduzierte Finerenon das Risiko der kardiovaskulären Mortalität [12].

Die derzeitigen Leitlinien enthalten noch keine Empfehlung zur Ergänzung der Therapie von Diabetes mellitus bei CKD. Während der Rekrutierung für FIGARO-DKD änderten sich die Empfehlungen zur T2DM-Therapie, sodass Subgruppen auch SGLT2i und GLP-1-RA erhielten. Subgruppenanalysen legten nahe, dass Finerenon auch bei diesen Patienten die kardiovaskuläre Mortalität reduziert [12], sodass eine Kombinationstherapie mit Finerenon, SGLT2i und gegebenenfalls GLP-1-RA möglicherweise von Vorteil sein könnte. Dies muss jedoch in weiteren Studien untersucht werden.

### SGLT2-Hemmer zur Therapie der chronischen Niereninsuffizienz

Mit Dapagliflozin wurde im August 2021 der erste SGLT2i zur Behandlung von Patienten mit CKD (unabhängig vom Vorhandensein eines T2DM) zugelassen. Grundlage hierfür waren die Ergebnisse der DAPA-CKD-Studie, in der 4304 Patienten mit oder ohne Diabetes mellitus und einer GFR zwischen 25 und 75 ml/min pro 1,73 m^2^ sowie einer UACR von 200 bis 5000 mg/g randomisiert Dapagliflozin (10 mg/Tag) oder Placebo erhielten [9]. Die Therapie mit Dapagliflozin erfolgte dabei unter maximal tolerierter Dosierung von ACEi oder ARB. Dapagliflozin reduzierte das Risiko für das Auftreten des kombinierten Endpunkts aus renal bedingter Mortalität, Progression zur terminalen Niereninsuffizienz oder Abnahme der GFR (um > 50 %) in 2,4 Jahren um 44 % [9]. Mit Dapagliflozin steht damit nun ein SGLT2i als zugelassene und wirksame Therapie zur Progressionshemmung der CKD zur Verfügung.

### Therapie der Begleiterkrankungen

Mit einer Verschlechterung der Nierenfunktion bei CKD können spezifische Komplikationen auftreten, deren konsequente Behandlung prognostisch relevant ist. Zu den möglichen Komplikationen zählen vor allem

- Hypervolämie,
- Störungen der Elektrolyte wie insbesondere Hyperkaliämie,
- metabolische Azidose,
- Störungen des Knochenstoffwechsels („chronic kidney disease-related mineral bone disorders“ [CKD-MBD]),
- Bluthochdruck,
- renale Anämie und
- eine Störung der Lipoproteine.

Im Folgenden soll darauf jeweils eingegangen werden.

#### Hypervolämie

Eine Hypervolämie ist bei CKD-Patienten mit einem erhöhten Risiko der Mortalität und Progression zur terminalen Niereninsuffizienz assoziiert [13]. In der KDIGO-Leitlinie wird eine Natriumrestriktion auf < 2 g/Tag empfohlen [3]. Zur diuretischen Therapie kommen Schleifendiuretika, Thiaziddiuretika und MRA infrage, wobei sich weder aus der KDIGO-Leitlinie [3] noch aus aktuellen Daten diesbezüglich eindeutige Empfehlungen ergeben [14]. Traditionell wurden bei fortgeschrittener CKD mit Volumenüberladung häufig Schleifendiuretika eingesetzt und weniger Thiaziddiuretika, da diese bei niedrigerer GFR als weniger wirksam galten. Dagegen zeigte die kürzlich publizierte CLICK-Studie, dass Chlortalidon bei CKD-Patienten mit einer mittleren GFR von 23 ml/min pro 1,73 m^2^ hocheffektiv zur Blutdrucksenkung ist [15]. In der Praxis ist eine individualisierte Einfach- oder Kombinationstherapie mit den genannten Diuretikaklassen zu empfehlen, wobei bei der Auswahl neben der klinischen Abschätzung des Volumenstatus auch der Zielblutdruck, die Serumelektrolyte und der Säure-Basen-Haushalt zu berücksichtigen sind.

#### Arterielle Hypertonie und Blutdruckeinstellung

In der KDIGO-Leitlinie zur Blutdruckeinstellung bei CKD von 2012 wurde eine Senkung auf < 130/80 mm Hg empfohlen. Im Jahr 2021 wurde die Leitlinie überarbeitet und gibt jetzt einen niedrigeren Zielblutdruck von < 120 mm Hg systolisch an [5]. Ausgenommen von der Empfehlung sind nierentransplantierte, dialysepflichtige und an Diabetes erkrankte Patienten, für die höhere Blutdruckziele gelten [5]. Grundlage für diesen Zielwert ist die SPRINT-Studie, in der SBD-Einstellungen auf < 120 mm Hg vs. < 140 mm Hg verglichen wurden [16]. Kardiovaskuläre Ereignisse und die Gesamtmortalität waren in der Gruppe mit dem niedrigeren Blutdruckziel von < 120 mm Hg signifikant reduziert [16]. Abweichend von dieser Empfehlung wird für Patienten mit Diabetes in den amerikanischen und europäischen Hypertonieleitlinien als Zielwert ein SBD < 130 mm Hg empfohlen [17, 18].

Zielblutdruckwerte für diabetische und nichtdiabetische CKD-Patienten sind aktuell unterschiedlich

In der SPRINT-Studie waren Patienten mit Diabetes mellitus ausgeschlossen. Die ACCORD-Studie untersuchte Zielblutdruckwerte bei Patienten mit Diabetes [19]. ACCORD zeigte keinen Vorteil einer strengeren SBD-Einstellung auf < 120 mm Hg im Hinblick auf kardiovaskuläre Ereignisse bei Patienten mit Diabetes [19]. Ob Gründe dafür im Studiendesign von ACCORD liegen, wird kontrovers diskutiert [20]. Die Zielblutdruckwerte für diabetische und nichtdiabetische CKD-Patienten sind daher nach aktuellen Empfehlungen unterschiedlich.

#### Metabolische Azidose

Die KDIGO-Leitlinie zum Management von CKD empfiehlt, dass Patienten mit CKD und einer Serumbikarbonatkonzentration < 22 mmol/l eine orale Bikarbonatsubstitution erhalten und normale Serumbikarbonatkonzentrationen angestrebt werden sollten [3]. Eine kürzlich erschienene Metaanalyse zeigte, dass der Ausgleich einer metabolischen Azidose durch orale Bikarbonatsubstitution bei CKD-Patienten mit einem niedrigeren CKD-Progressions-Risiko assoziiert ist [21]. Allerdings ist die Evidenzlage insgesamt schwach.

#### Hyperkaliämie

Hyperkaliämie ist ein häufiges Problem bei Patienten mit CKD. Dies ist begründet in der verminderten renalen Kaliumexkretion und wird durch kaliumreiche Ernährung oder hyperkaliämieinduzierende Komedikation (ACEi/ARB/MRA) potenziert [22]. Die KDIGO-Leitlinie zum Management von CKD empfiehlt, dass Patienten mit CKD eine Ernährungsberatung für eine kaliumangepasste Kost erhalten sollten [3]. Eine medikamentöse Empfehlung zur Behandlung der chronischen Hyperkaliämie bei Patienten mit CKD enthält die KDIGO-Leitlinie allerdings nicht. Die britische Leitlinie hingegen empfiehlt bereits den Einsatz von oralen Kaliumbindern bei Patienten mit CKD im Stadium 3b bis 5 (nicht dialysepflichtig) und persistierender Hyperkaliämie > 6 mmol/l unter ACEi/ARB-Therapie [23]. Neben den seit Längerem verfügbaren Kaliumbindern Kalziumpolystyrolsulfonat (CPS-Pulver) und Natriumpolystyrolsulfonat sind kürzlich Patiromer und Natrium-Zirkonium-Zyklosilikat zugelassen worden. Eine Metaanalyse zu den vier Kaliumbindern hat eine schwache Evidenz für ihren Einsatz bei der Behandlung der Hyperkaliämie bei CKD gezeigt. Die verfügbaren Kaliumbinder können die Kaliumwerte bei Patienten mit CKD senken, ein günstiger Einfluss auf die Mortalität oder CKD-Progression ist allerdings bisher nicht gezeigt [24].

#### Metabolische Störungen des Knochenstoffwechsels

Die KDIGO-Leitlinie zum CKD-Management von 2012 empfiehlt die Bestimmung von Kalzium, Phosphat, intaktem Parathormon (iPTH) und alkalischer Phosphatase ab einer GFR < 45 ml/min pro 1,73 m^2^ [3]. Hier ist mit dem Auftreten eines sekundären Hyperparathyreoidismus zu rechnen, der durch ein komplexes Zusammenspiel aus Vitamin-D-Mangel, erhöhtem „fibroblast growth factor 23“ (FGF23), Hypokalzämie und Hyperphosphatämie charakterisiert ist. Eine eigene Leitlinie zu CKD-MBD wurde von der KDIGO 2017 überarbeitet [25]. Sie empfiehlt als erste therapeutische Maßnahme eine Normalisierung der Serumphosphatwerte. Für eine präventive phosphatsenkende Therapie bei Patienten mit CKD G3b–G4 und noch normalen Phosphatwerten gibt es aktuell keine Evidenz [25]. Bei persistierender iPTH-Erhöhung sollten zunächst eine Hyperphosphatämie, eine Hypokalzämie und ein Vitamin-D-Mangel ausgeschlossen werden [25].

#### Renale Anämie

Eisen- und Erythropoetin(EPO)-Mangel mit erniedrigten Retikulozyten sind die Hauptursache einer Anämie bei Patienten mit CKD. Bei der Therapie gilt das Prinzip „Eisen vor EPO“. So wird in der KDIGO-Leitlinie bei CKD-Patienten mit symptomatischer Anämie und einer Transferrinsättigung < 30 % sowie einem Ferritinspiegel < 500 µg/l eine vorzugsweise intravenöse Eisensubstitution empfohlen. Der Einsatz von EPO sollte bei CKD-Patienten mit einem Hämoglobin(Hb)-Wert < 10 g/dl nach Abwägung der Symptome, der Hb-Dynamik und des Ansprechens auf einen Ausgleich des Eisenmangels erfolgen. Für den Hb-Zielwert unter EPO gilt nicht „je höher, desto besser“. Im Gegenteil, in vielen Studien zum Ziel-Hb unter EPO-Substitution waren Hb-Werte > 11 g/dl mit einer erhöhten Rate an kardiovaskulären Ereignissen und malignen Erkrankungen sowie einer erhöhten Mortalität assoziiert [26]. Die KDIGO-Leitlinie zu renaler Anämie empfiehlt daher, den Hb-Wert unter EPO-Therapie nicht dauerhaft über 11,5 g/dl zu halten [27].

HIF-Prolyl-Hydroxylase-Inhibitoren (*HIF* hypoxieinduzierbarer Faktor) wie Daprodustat, Vadadustat und Roxadustat sind neue Substanzen, die über eine Erhöhung des endogenen EPO-Spiegels wirken. Als oral verfügbare Therapeutika stellen sie eine Alternative zu den subkutan oder intravenös zu verabreichenden EPO-Derivaten dar und erscheinen daher insbesondere zur Anämiebehandlung bei nichtdialysepflichtigen Patienten geeignet.

#### Dyslipoproteinämie

Störungen des Lipidmetabolismus sind häufig bei CKD und ein entscheidender Faktor für das hohe kardiovaskuläre Risiko von Patienten mit CKD [28]. Die KDIGO hat eine eigene Leitlinie für Störungen des Lipidmetabolismus bei CKD publiziert und empfiehlt mit Ausnahme von Dialysepatienten bei allen CKD-Patienten > 50 Jahre eine Statintherapie [29]. Bei Patienten < 50 Jahren sollte eine Statintherapie erfolgen, wenn ein zusätzlicher kardiovaskulärer Risikofaktor, wie Diabetes mellitus, vorliegt [29].

### Anpassung der Medikamentendosis

Die KDIGO-Leitlinie zum Management von CKD empfiehlt, die Indikation einer nephrotoxischen Medikation sorgfältig zu prüfen und die Dosierung an die Nierenfunktion anzupassen. Bei Patienten mit einer GFR < 60 ml/min pro 1,73 m^2^, die ernsthaft akut erkrankt sind, empfiehlt die Leitlinie, potenziell nephrotoxische Medikamente, wie ACEi, ARB, Spironolacton, Lithium oder Digoxin, zu pausieren [3]. Diese „Sick-day-Pausierung“ wird auch bei SGLT2i empfohlen.

### Vorbereitung Nierenersatzverfahren

Die 2015 überarbeitete KDIGO-Praxisleitlinie zur Hämodialyse empfiehlt, dass Patienten mit einer GFR < 30 ml/min pro 1,73 m^2^ bereits über mögliche Nierenersatzverfahren (Hämodialyse, Peritonealdialyse, Nierentransplantation als Lebendspende oder von hirntoten Organspendern) aufgeklärt werden sollten, um rechtzeitig die notwendigen Vorbereitungen (Dialysezugang, Transplantationsabklärung) treffen zu können [30].

## Fazit für die Praxis

- Die Diagnose chronische Nierenerkrankung („chronic kidney disease“ [CKD]) kann gestellt werden, wenn ein struktureller Nierenschaden vorliegt oder die glomeruläre Filtrationsrate (GFR) für ≥ 3 Monate reduziert ist.
- CKD wird in die Stadien G1–G5 anhand der GFR und A1–A3 anhand der Albuminurie („urinary albumin-creatinine ratio“ [UACR]) eingeteilt. Die Einteilung ist prognostisch relevant.
- Der Großteil der CKD-Patienten mit GFR > 60 ml/min pro 1,73 m^2^ wird ausschließlich hausärztlich oder allgemeininternistisch und nicht nephrologisch betreut.
- Antihypertensive Therapeutika der Wahl bei CKD sind ACEi oder ARB. Das Ziel für den systolischen Blutdruck ist < 120 mm Hg.
- Mit Dapagliflozin ist ein zugelassenes und wirksames Therapeutikum zur Hemmung der CKD-Progression verfügbar.
- Für Patienten mit Diabetes mellitus Typ 2 und CKD wurde mit Finerenon ein weiteres Therapeutikum mit günstigem Einfluss auf die CKD-Progression zugelassen.
- Hypervolämie, Hyperkaliämie, metabolische Azidose, arterieller Hypertonus, Störungen des Knochenstoffwechsels, renale Anämie und Dyslipoproteinämie sind wichtige Begleiterkrankungen der CKD, die konsequent therapiert werden sollten.
